# Abnormal Skeletal Growth in Adolescent Idiopathic Scoliosis Is Associated with Abnormal Quantitative Expression of Melatonin Receptor, MT2

**DOI:** 10.3390/ijms14036345

**Published:** 2013-03-19

**Authors:** Annie Po-yee Yim, Hiu-yan Yeung, Guangquan Sun, Kwong-man Lee, Tzi-bun Ng, Tsz-ping Lam, Bobby Kin-wah Ng, Yong Qiu, Alain Moreau, Jack Chun-yiu Cheng

**Affiliations:** 1 Department of Orthopaedics and Traumatology, The Chinese University of Hong Kong, Hong Kong, China; E-Mails: anniepyim@gmail.com (A.P.Y.); benson914@gmail.com (H.Y.); sunguangquan1981@163.com (G.S.); tplam@ort.cuhk.edu.hk (T.L.); bobng@ort.cuhk.edu.hk (B.K.N.); 2 Lee Hysan clinical research laboratory, The Chinese University of Hong Kong, Hong Kong, China; E-Mail: simonlee@cuhk.edu.hk; 3 School of Biomedical Science, The Chinese University of Hong Kong, Hong Kong, China; E-Mail: tzibunng@cuhk.edu.hk; 4 Spine Surgery, The Affiliated Drum Tower Hospital of Nanjing University Medical School, Nanjing 210000, China; E-Mail: scoliosis2002@sina.com; 5 The Joint Scoliosis Research Center of the Chinese University of Hong Kong and Nanjing University, Hong Kong, China; 6 Research Center, Sainte-Justine Hospital, University of Montreal, Montreal, QC H3T 1C5, Canada; E-Mail: alain.moreau@recherche-ste-justine.qc.ca

**Keywords:** idiopathic scoliosis, melatonin, receptors, osteoblasts

## Abstract

The defect of the melatonin signaling pathway has been proposed to be one of the key etiopathogenic factors in adolescent idiopathic scoliosis (AIS). A previous report showed that melatonin receptor, MT2, was undetectable in some AIS girls. The present study aimed to investigate whether the abnormal MT2 expression in AIS is quantitative or qualitative. Cultured osteoblasts were obtained from 41 AIS girls and nine normal controls. Semi-quantification of protein expression by Western blot and mRNA expression by TaqMan real-time PCR for both MT1 and MT2 were performed. Anthropometric parameters were also compared and correlated with the protein expression and mRNA expression of the receptors. The results showed significantly lower protein and mRNA expression of MT2 in AIS girls compared with that in normal controls (*p* = 0.02 and *p* = 0.019, respectively). No differences were found in the expression of MT1. When dichotomizing the AIS girls according to their MT2 expression, the group with low expression was found to have a significantly longer arm span (*p* = 0.036). The results of this study showed for the first time a quantitative change of MT2 in AIS that was also correlated with abnormal arm span as part of abnormal systemic skeletal growth.

## 1. Introduction

Adolescent idiopathic scoliosis (AIS) is a complex three-dimensional structural deformity of the spine that occurs most commonly in girls between ages 10 and 16 with a prevalence rate of 2%–4% [1,2]. Complications, such as cardiopulmonary failure, back pain and significant cosmetic problems could occur in severe cases with curve progression. Despite intense research in the field, the etiology and etiopathogenesis of AIS has not been fully elucidated. In addition to genetics, abnormal neurophysiological function and abnormal asynchronous skeletal growth in AIS [1,2], studies on melatonin deficiency and its related receptor and signaling pathway dysfunction have attracted much attention in recent years.

Melatonin, or *N*-acetyl-5-methoxytryptamin, is a hormone mainly synthesized and secreted by the pineal gland and other organs and tissues [3,4]. Apart from the well-known function in controlling circadian rhythm [5], melatonin is also found to be involved in blood pressure regulation [6], immune function [7], scavenging of free radicals [8,9], control of tumor growth [10] and bone growth [11,12].

It is suggested that melatonin, besides its direct effect, such as radical scavenging [9], could exert its function via various binding sites, such as RZR/ROR isoforms, as nuclear receptors [13], mitochondrial binding sites [8] and also membrane receptors [14]. Melatonin receptor MT1 and MT2 are two types of membrane receptors, which have been cloned and well defined in humans [15,16]. Both of them belong to the family of G-protein coupled receptors (GPCR) [15,16] and are present in the retina [17–19], brain [20,21] and other tissues [22–25]. The local effect of melatonin on cells through melatonin receptors is mediated by different pathways and secondary messengers, depending on the cell type. In human osteoblasts, the best recognized signaling pathway of melatonin receptors is the cAMP-dependent pathway [26]. The activation of MT1 or MT2 by melatonin can inhibit adenylate cyclase activity [26], resulting in the inhibition of forskolin-induced cyclic AMP formation and leads to a decrease in activated protein kinase A [27] and subsequent downstream signaling.

Melatonin was found to play an important role in modulating bone growth and bone mineralization in many *in vitro* studies [28–30]. Melatonin deficiency is associated with a low bone mass [31], which was shown to be a systemic phenomenon in AIS [32,33]. Pinealectomy-induced melatonin deficiency has been demonstrated in different animal models [34–40]. The effect of deficiency could be rectified by melatonin pellet implantation [41]. These observations led to the hypothesis that melatonin is associated with osteopenia and the occurrence of AIS. However, studies on the melatonin levels in AIS patients have yielded inconsistent results [42–44]. More recent studies have been extended to melatonin receptors and the downstream signaling pathway through which melatonin produces most of its biological effects [45,46].

Recently, our group has observed an association of the occurrence of AIS with a polymorphism (SNP) in the promoter region of the MT2 (or MTNR1B) gene [47]. The study by Moreau *et al*. [45] suggested the presence of dysfunction of melatonin signaling in osteoblasts from AIS patients, and Azeddine *et al.* proposed a molecular classification of AIS patients according to their different cellular response towards melatonin [46]. In another study, we also found an abnormal proliferative response of osteoblasts to melatonin together with the observation of undetectable MT2 in a small subgroup of AIS [48,49]. We speculated that although MT2 is expressed at a low level in human beings [50]; its abnormal expression in AIS patients might directly affect the melatonin signaling pathway and, hence, the physiological modulating effect of melatonin. In the present study, the quantitative expression of MT1 and MT2 was evaluated at both protein and mRNA levels, and its relationship with the anthropometric parameters of skeletal growth was analyzed.

## 2. Results and Discussion

### 2.1. Semi-Quantification of Protein Expression Levels of MT1 and MT2 in Osteoblast

**Figure 1:**
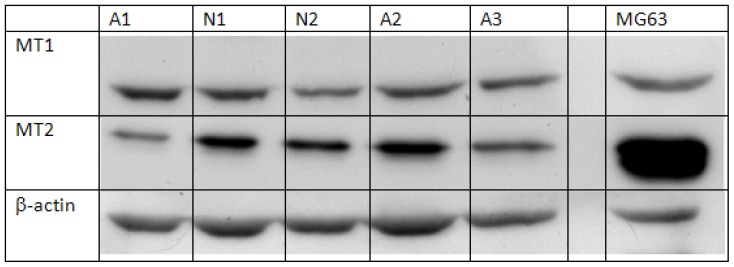
Representative image of protein expression of melatonin receptors in osteoblasts. Cells isolated from normal controls were cultured until confluence. Cells were then collected and lysed for analysis of protein expression of melatonin receptor MT1 and MT2. Beta-actin was used as an internal control, and protein from the cell line MG63 was used for positive control. N = normal control; A = AIS subject.

The Western blot signals of melatonin receptors of normal controls and AIS patients are shown in Figure 1. All of the normal controls showed the presence of MT1 and MT2. Comparison between the AIS group and control group disclosed that the intensity of MT1 was similar between the two groups (*p* = 0.85) using independent sample Student’s *t*-test. (Table 1) However, the expression level of MT2 in the AIS group was significantly lower than that in the control group (*p* < 0.01).

In a previous study by Man *et al.*[49], it was observed, with a small sample size, that MT2 was not detectable in the osteoblasts of a group of AIS patients, which at the same time, showed an abnormal proliferation in response to melatonin. Results from the functional assay performed by Moreau *et al.*[45] also suggested that there was a differential melatonin signaling dysfunction in AIS patients, which might be due to abnormal coupling of G-protein. Melatonin is essential in bone function, such as proliferation and differentiation of osteoblasts [28,30,31], and melatonin receptors play an important role in the mediation of melatonin signaling [29,51,52]. It has been shown that a MT2 antagonist, 4-phenyl-2-propionamidotetraline (4P-PDOT), could inhibit melatonin-stimulated proliferation in the control human osteoblasts [48].

The present study employing a larger sample size supported the postulation of Man *et al.*[49] (41 AIS and nine control in this study *vs.* 11 AIS and eight control in Man *et al.*) that there is an abnormality in melatonin receptor expression, which might account for the melatonin signaling dysfunction in the osteoblasts. This study also further revealed a lower quantitative expression of MT2 rather than the absence of MT2, as suggested by Man *et al.*[49]. The discrepancy between the results of the two studies could be explained by differences in the experimental procedures, such as the choice of antibodies, the method and time of blotting and signal development during the assessment of protein expression.

### 2.2. Quantification of mRNA Expression Levels of MT1 and MT2 in Osteoblast

**Figure 2:**
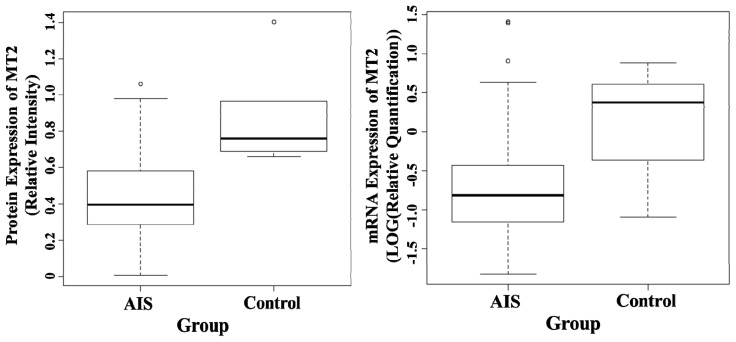
Boxplot of protein expression (left) and mRNA expression of melatonin receptor 2, MT2 (right). The protein level was measured by Western Blot, and beta-actin was used as an internal control. The mRNA level was measured by RT-PCR using TaqMan with a pre-made primer. The results were quantified using a value relative to a control sample. The bottom and top of the box are the twenty fifth and seventy fifth percentile and, the band in the box is the median. The ends of the whiskers represent the lowest datum within 1.5 interquartile range (IQR) of the lower quartile and the highest datum within 1.5 IQR of the upper quartile. Data not included between the whiskers are plotted as circles.

The comparison of mRNA expression levels of melatonin receptors between AIS and control groups is shown in Table 1. The mRNA expression level of MT2 of AIS patients was significantly lower than the control (*p* = 0.019) (Figure 2), while the mRNA expression level of MT1 was similar between the two groups (*p* = 0.707) when compared by Student’s *t*-test.

In contrast to the previous observation by Man *et al.*[49] using Western blot, results from real-time PCR employed in the present study ruled out the total absence of MT2 expression in AIS girls, as it allowed the detection of any quantitative difference in the mRNA expression level. The lower mRNA expression level of MT2 in AIS girls indicated that, other than possible factors, such as protein stability and the process of translation, the abnormal protein expression level might be due to upstream events, such as gene expression. Our previous study by Qiu *et al.*[47] found the association of a single nucleotide polymorphism (SNP) in the promoter region of the MT2 (or MTNR1B) gene with the occurrence of AIS. It is likely that this polymorphism could affect the gene expression of MT2, thus accounting for the lower mRNA expression level of MT2 found in AIS patients in the current study. The results of Qiu’s study could not be reproduced by a few related studies [53–56], which might partly be explained by the different ethnic populations [54–56] recruited for the studies. It is recognized that the ratio of combinations of the SNP of interest is different among different ethnic groups (Hapmap). In the study of Shyy *et al*. [53], only 180 AIS patients and 180 normal controls were recruited, a much smaller number than the sample size of 814 AIS patients and 651 controls used in the study of Qiu *et al*. [47]. These may explain the negative results found by other research groups. Besides mutation or SNP of the gene, other upstream events, such as transcription, pre-mRNA processing (splicing, polyadenylation) and mRNA stability could all affect the level of mRNA. Slominiski *et al.*[57] reported the presence of aberrantly spliced mRNA of MT2 in the pituitary, which could not be translated correctly into protein and might not be detected by PCR, because of the specificity of the primer. This is one of the possible explanations for the lower mRNA level in AIS. However, not all the AIS patients in this study showed a low expression of MT2, implying the heterogeneity of the disease and the presence of etiologies other than melatonin signaling dysfunction. The heterogeneous nature of AIS may also explain why associated common variants near melatonin receptor genes have not yet been identified in association studies [56].

### 2.3. Evaluation and Correlation of Anthropometric Parameters with Melatonin Receptors Expression Level

Table 2 shows a comparison of body weight, arm span and body mass index (BMI) between AIS patients and the normal population. AIS patients had a lower body weight, a significantly lower BMI (*p* < 0.01) and a longer arm span (*p* < 0.01).

AIS patients were divided into two subgroups (normal and low-expression) according to their mRNA expression level of melatonin receptors using the minimum value of normal controls as a cut-off point. Arm span was used as an indicator of longitudinal growth of body height, as it had been found to be a more reproducible and reliable clinical anthropometric parameter than the formulae-based corrected body height [58–60]. Table 3 presents a comparison of anthropometric parameters between the two groups for MT1 and MT2. For MT1, the *z*-scores between the normal and low-expression groups were similar, and there were no significant differences in all the anthropometric parameters. Patients with a low expression level of MT2 had a significantly longer arm span (*p* = 0.036), but there were no significant differences in body weight and BMI.

Similar to the results from previous studies [61–63], a significant percentage of AIS patients was found to have longer arm span, lower body weight and a reduced BMI, as shown above. However, this study for the first time demonstrated that AIS patients with a lower MT2 mRNA expression level exhibited a significantly longer arm span than patients with a normal expression level. It is speculated that melatonin signaling dysfunction due to abnormal melatonin receptor might be associated with abnormal modulation and regulation of skeletal bone growth in AIS. Melatonin has been reported to play a role in bone growth and metabolism [11,12,64]. The hormone also has an effect on secretion of growth hormone, the main regulator of growth during puberty.

## 3. Experimental Section

### 3.1. Subjects Recruitments

Forty-one girls (aged 9 to 19, mean age: 15.2 ± 2.0) with severe AIS were recruited from the Joint Scoliosis Research Center of the Chinese University of Hong Kong and Nanjing University. The diagnosis of idiopathic scoliosis was by clinical examination and standard standing posteroanterior X-ray. Patients, after identifying scoliosis of congenital, neuromuscular, metabolic etiology, skeletal dysplasia, known endocrine or connective tissue abnormalities or with ongoing medications, were excluded. Trabecular bones of the patients were obtained intra-operatively from the iliac crests.

Nine non-scoliotic normal controls (8 female and 1 male) aged between 12 and 24 (mean age: 17.5 ± 4.7) were recruited. Bone biopsies were collected from the bone tunnels of patients undergoing anterior cruciate ligament reconstruction (3 cases) or the non-affected sites of patients with degenerative spine undergoing spinal fusion surgery (6 cases). The absence of scoliosis and other bone metabolic diseases were confirmed by experienced orthopedic surgeons before the operation. The research protocol had been approved by the Clinical Research Ethics Committee of the University. Informed consents to collect bone biopsy during operation were acquired from patients before the surgery.

### 3.2. Cell Isolation and Osteoblast Culture

Bone biopsies were first washed with plain DMEM containing 10% penicillin-streptomycin-neomycin (PSN) antibiotic mixture (Invitrogen, Carlsbad, NM, USA) to avoid any contamination. Trabecular bones from the biopsies were cut and minced into small pieces with a sharp bone cutter under sterile conditions. The fragments were plated onto a 6-well culture plate (Corning, New York, NY, USA) and cultured in DMEM (Invitrogen) supplemented with 10% fetal bovine serum (FBS) (Invitrogen) and 1% PSN (Invitrogen). The culture was maintained at 37°C in a humidified atmosphere of 5% CO_2_. The medium was renewed every 2–3 days. Cells were harvested by the addition of 0.25% trypsin (Invitrogen) upon reaching 90% confluence for subculture. The second passage of cells was used for the following assessments.

### 3.3. Semi-Quantification of Protein Expression of MT1 and MT2 in Osteoblasts

Osteoblasts (1 × 10^6^) were lysed in RIPA buffer (Sigma, St. Louis, MO, USA) containing 1 mM protease inhibitor cocktail (Sigma,) and 1mM phenylmethylsulfonyl fluoride (PMSF) (Sigma). Protein was collected from the lysed cells, and the protein concentration was determined by Bradford assay. The protein expression of melatonin receptor was determined by Western blot. Extracted protein (30 μg) was denatured and separated by SDS-PAGE (Bio-Rad, Hercules, CA, USA). For each gel, the same amount of protein extracted from MG63 cells (human osteoblastic cells expressing both MT1 and MT2 [65]) was loaded to serve as a positive control. The gel with separated proteins was removed and electroblotted for 20 min at 500 mA onto a nitrocellulose membrane (Hybond-ECL; GE Healthcare, Bucks, UK) by using a semi-dry method. The membrane was blocked with 5% fat-free milk and probed with the selected primary antibodies (goat IgG anti-human MT1: Abcam, Cambridge, England; rabbit IgG anti-human MT2: Novus Biologicals, Littleton, CA, USA). The probed membrane was then incubated with selected horseradish peroxidase-conjugated secondary antibody (goat anti-rabbit IgG: Abcam, Cambridge, England; donkey anti-goat IgG: Santa Cruz, Santa Cruz, CA, USA), and the immunocomplex was visualized using ECL Western Blotting Detection Reagents (GE Healthcare, Bucks, UK) on a medical X-ray film (Fujifilm, Tokyo, Japan).

To control and correct for protein loading error and allow comparison between bands, beta-actin was used as an internal control. The same membrane, after analysis using the first antibody, was incubated with an anti-actin antibody (Abcam, Cambridge, UK) and then with horseradish peroxidase-conjugated donkey anti-goat secondary antibody (Santa Cruz, Santa Cruz, CA, USA). The immunocomplex was then visualized, as previously mentioned. Band intensity was obtained by an image analysis software, ImageJ (v.1.43, National Institute of Health Maryland, Bethesda, MD, USA). The corrected intensity of the expression of MT1 and MT2 of each sample was calculated by adjusting against the signal intensity of beta-actin. All results were confirmed in duplicate.

### 3.4. Quantification of mRNA Expression of MT1 and MT2 in Osteoblast

Osteoblast cells (5 × 10^5^) were used for mRNA extraction with a QIAGEN RNeasy Extraction Kit (Qiagen, Hilden, Germany), according to manufacturer’s instructions. The mRNA concentration of each sample was determined by Nanodrop 2000 (Thermo Fisher Scientific, Beverly, MA, USA). Total RNA (2 μg) of each sample was converted to cDNA by transcription reaction with 1 μL of 10 mM dNTP (Promega, Fitchburg, WI, USA), 30 ng of oilgo dT (Invitrogen) and 20 units of RNase inhibitor (Invitrogen). The real-time PCR reaction was performed with TaqMan Gene Expression Assay (Applied Biosystem, Foster City, CA, USA). A pre-made TaqMan Gene Expression Assay (Assay id: Hs00195567_m1*) and a custom-made assay (Assay id: AJWR10X) flanking the *N*-terminal extracellular domain of MT2 was used for MT1 and MT2, respectively. A pre-made assay of GAPDH (Assay id: Hs03929097_g1), a house-keeping gene, was used as an endogenous control to correct for loading error and difference in cDNA concentration between samples. The PCR reaction was performed on a 7900HT Fast Real-Time PCR System (Applied Biosystem) using a 384-well plate, according to the manufacturer’s guidelines. Relative quantitation of mRNA of melatonin receptors was calculated using RQ manager (Applied Biosystem) and SDS software (Applied Biosystem). All samples were run in duplicate, and a normal control sample was used as a calibrator. The RQ values were expressed in log to obtain a normal distribution for analysis.

### 3.5. Evaluation and Correlation of Anthropometric Parameters with Melatonin Receptors Expression

The anthropometric parameters, including body weight (BW), body mass index (BMI) and arm span, were measured with standard methods. The body weight was measured on an electronic balance (Soechnle, Germany), and the subjects were required to wear light garments, with shoes removed, during the measurement. Accuracy was taken to the closest 0.1 kg. For measuring the arm span, subjects were asked to fully stretch their arms horizontally against a wall-mounted tape. Accuracy was taken to the closest 0.1 cm. Body mass index (BMI) was calculated by dividing body weight (kg) by the square of arm span (m2). The measurements were made with reference to the database of 525 subjects (normal healthy adolescent girls) at our center and converted to age-matched *z*-scores for comparison between subjects of different ages. For subgroup analysis, the AIS patients were separated into two groups according to their mRNA expression level of melatonin receptor (normal or low), using the minimum value of expression level of normal controls as a cut-off point. The *z*-scores of different parameters were compared between the two groups.

### 3.6. Data Analysis

The normalized values of band intensity from Western blot and the log of RQ values from RT-PCR of AIS subjects and normal controls were analyzed by Independent Sample Student’s *t*-test. The *z*-scores of anthropometric parameters of AIS patients were compared against the normal adolescent girls by one sample *t*-test to test for deviation from zero. An independent sample Student’s *t*-test was used to compare the *z*-scores between groups of AIS patients with different expression levels of melatonin receptors. The data were expressed as the mean ± SD. The difference was considered statistically significant when *p* was less than 0.05. Statistical analysis software SPSS ver 16.0 (SPSS Inc., Chicago, IL, USA) was used for statistical analysis.

## 4. Conclusions

In this study, a quantitative difference in both the protein and mRNA expression levels of MT2 was found between AIS patients and normal controls. The expression level of MT2 was significantly lower in girls with AIS. It is also found that AIS girls with low MT2 expression had a longer arm span. Although the normal subjects recruited consisted of both males and females and the age spectrum was older than that of AIS patients, due to the difficulty to obtain bone biopsies for practical and ethical reasons, the present study provided new important observation relating the role of melatonin and its receptors and signaling pathway to the etiopathogenesis of AIS. To find out how the MT2 expression level could affect osteoblast function and, hence, bone growth and bone quality, further animal studies and functional cellular and molecular genetic tests would be required. The exact mechanism of how the quantitative changes of MT2 in AIS affect osteoblast function warrants further investigations, with the hope of understanding better the role of melatonin signaling in skeletal bone growth and the implication of arm span as an prognostic indicator in severe AIS.

## Figures and Tables

**Table 1:** Expression of melatonin receptor MT1 and MT2 in osteoblasts.

Expression of		AIS (*n* = 41) (mean ± SD)	Control (*n* = 9) (mean ± SD)	*p*-value
Protein (Relative intensity)	MT1	0.797 ± 0.199	0.793 ± 0.322	0.978
	MT2	0.429 ± 0.276	0.881 ± 0.317	<0.01 *
mRNA [Log (Relative quantification)]	MT1	−0.378 ± 0.258	−0.343 ± 0.225	0.707
	MT2	−0.627 ± 0.742	0.129 ± 0.70	0.019 *

The protein and mRNA expression levels of melatonin receptor MT1 and MT2 between AIS and control were compared by Student’s *t*-test,

* *p* < 0.05. All data were expressed as the mean ± SD. The protein level was expressed in relative intensity after correction by beta-actin.

**Table 2:** Clinical evaluation of AIS patients. The *z*-score of body weight (BW), body mass index (BMI) and arm span of AIS patients were compared against the normal population by one sample Student’s *t*-test using zero as the reference value.

	AIS patients (*n* = 38) mean ± SD †	*z*-score mean ± SD	*p*-value
BW (kg)	46.46 ± 6.36	−0.15 ± 0.91	0.29
BMI	18.37 ± 2.16	−0.45 ± 0.15	<0.01 *
Arm Span (cm)	158.96 ± 6.13	0.41 ± 0.83	<0.01 *

All data were expressed as the mean ± SD,

* *p* < 0.01.

† The mean values were not adjusted for age.

**Table 3:** Correlation of clinical anthropometric parameters of AIS patients with mRNA expression of melatonin receptor MT1 and MT2. The AIS patients were divided into groups according to their mRNA expression level of melatonin receptor MT1 or MT2 using the minimum value of normal controls as the cut-off point.

Melatonin receptor MT1 (*n* = 38)			
Group	Normal expression (*n* = 32)	Low expression (*n* = 6)	*p*-value
*z*-score of			
BW	−0.136 ± 0.98	−0.070 ± 0.62	0.875
BMI	−0.429 ± 0.98	−0.384 ± 0.67	0.915
Arm span	0.412 ± 0.92	0.471 ± 0.28	0.768
Expression of mRNA (Log (relative quantification))	−0.311 ± 0.18	−0.751 ± 0.078	<0.01
Age	15.3 ± 2.1	15.2 ± 1.5	0.940
**Melatonin receptor MT2 (*n*= 38)**			
**Group**	**Normal expression (*n*= 27)**	**Low expression (*n*= 11)**	***p*-value**
***z*-score of**			
BW	−0.223 ± 0.83	0.112 ± 1.13	0.318
BMI	−0.405 ± 0.83	−0.464 ± 1.18	0.861
Arm Span	0.238 ± 0.79	0.870 ± 0.86	0.036 *
Expression of mRNA (Log (relative quantification))	−0.367 ± 0.72	1.311 ± 0.21	<0.01
Age	15.1 ± 1.9	15.7 ± 2.0	0.414

Their anthropometric parameters [body weight (BW), body mass index (BMI) and arm span] were compared by Student’s *t*-test,

* *p* < 0.05. All data were expressed as the mean ± SD.
